# Methods to Evaluate Changes in Mitochondrial Structure and Function in Cancer

**DOI:** 10.3390/cancers15092564

**Published:** 2023-04-29

**Authors:** Brittany P. Rickard, Marta Overchuk, Vesna A. Chappell, Mustafa Kemal Ruhi, Prima Dewi Sinawang, Tina Thuy Nguyen Hoang, Demir Akin, Utkan Demirci, Walfre Franco, Suzanne E. Fenton, Janine H. Santos, Imran Rizvi

**Affiliations:** 1 Curriculum in Toxicology & Environmental Medicine, School of Medicine, University of North Carolina at Chapel Hill, Chapel Hill, NC 27599, USA; 2 Joint Department of Biomedical Engineering, University of North Carolina at Chapel Hill, Chapel Hill, NC, and North Carolina State University, Raleigh, NC 27695, USA; 3 Mechanistic Toxicology Branch, Division of Translational Toxicology, National Institute of Environmental Health Sciences, Research Triangle Park, NC 27709, USA; 4 Institute of Biomedical Engineering, Boğaziçi University, Istanbul 34684, Turkey; 5 Canary Center at Stanford for Cancer Early Detection, Department of Radiology, School of Medicine, Palo Alto, CA 94304, USA; 6 Department of Chemical Engineering, Stanford University, Stanford, CA 94305, USA; 7 Department of Biomedical Engineering, University of Massachusetts Lowell, Lowell, MA 01854, USA; 8 Center for Cancer Nanotechnology Excellence for Translational Diagnostics (CCNE-TD), School of Medicine, Stanford University, Stanford, CA 94305, USA; 9 Lineberger Comprehensive Cancer Center, University of North Carolina School of Medicine, Chapel Hill, NC 27599, USA; 10 Center for Environmental Health and Susceptibility, University of North Carolina at Chapel Hill, Chapel Hill, NC 27599, USA

**Keywords:** mitochondrial methods, bioenergetics, mitochondrial dysfunction, reactive species, mitochondrial function, mitochondrial structure, extracellular vesicles

## Abstract

**Simple Summary:**

Mitochondria, which play crucial roles in energy production and in maintaining cellular homeostasis, are potential therapeutic targets in a variety of diseases including cancer. This review discusses methods to evaluate the structural and functional parameters of mitochondrial health in the context of tumors and cancer cells. These methods include microscopy to examine mitochondrial morphology, PCR to measure changes in mitochondrial DNA content, and various technologies, such as Seahorse analyzers and the Clark electrode, to measure metabolic function. Other functional parameters, such as membrane potential and oxidative stress levels, can be measured using commercially available dyes and assays. By leveraging these techniques, insights can be gained into the biology, metastatic potential, and response to therapy of tumors, ultimately leading to improved treatment plans.

**Abstract:**

Mitochondria are regulators of key cellular processes, including energy production and redox homeostasis. Mitochondrial dysfunction is associated with various human diseases, including cancer. Importantly, both structural and functional changes can alter mitochondrial function. Morphologic and quantifiable changes in mitochondria can affect their function and contribute to disease. Structural mitochondrial changes include alterations in cristae morphology, mitochondrial DNA integrity and quantity, and dynamics, such as fission and fusion. Functional parameters related to mitochondrial biology include the production of reactive oxygen species, bioenergetic capacity, calcium retention, and membrane potential. Although these parameters can occur independently of one another, changes in mitochondrial structure and function are often interrelated. Thus, evaluating changes in both mitochondrial structure and function is crucial to understanding the molecular events involved in disease onset and progression. This review focuses on the relationship between alterations in mitochondrial structure and function and cancer, with a particular emphasis on gynecologic malignancies. Selecting methods with tractable parameters may be critical to identifying and targeting mitochondria-related therapeutic options. Methods to measure changes in mitochondrial structure and function, with the associated benefits and limitations, are summarized.

## 1. Introduction

The mitochondrion is an organelle present in almost all eukaryotic cells. In mammalian cells, mitochondria generate up to 90% of adenosine triphosphate (ATP), a universal source of chemical energy, through a process called oxidative phosphorylation (OXPHOS) [1,2]. As a byproduct of OXPHOS, mitochondria also generate reactive oxygen species (ROS) that can induce oxidative damage or may be involved in signaling. In addition to their pivotal role in cellular bioenergetics, metabolic pathways occurring in mitochondria produce metabolites that are important for a variety of processes, including the synthesis of lipids, amino acids, and nucleotides, as well as the maintenance of the epigenome [3,4]. Additional processes affected by mitochondrial function and signaling include cell proliferation and apoptosis [5]. Given the central role of mitochondria in cellular metabolism, bioenergetics, and signaling, evaluation of mitochondrial dysfunction as a mechanistic driver of cancer pathogenesis is well-justified. Likewise, deregulated cellular energetics are among the hallmarks of cancer, and evidence suggests that mitochondrial dysfunction may be an important factor in cancer development and progression [6,7,8]. The degree of mitochondrial dysfunction is associated with disease aggressiveness, highlighting the mitochondrion as an important diagnostic and therapeutic target [9,10,11].

**Figure 1:**
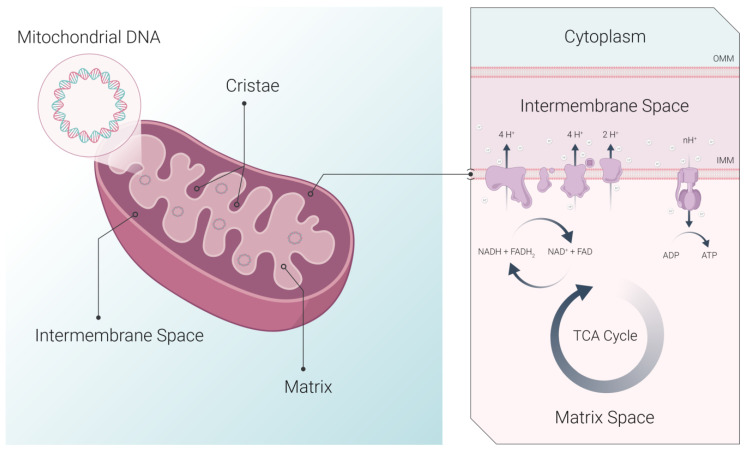
Schematic representation of the structure of a mitochondrion and select features of the matrix and intermembrane space (inset). Tricarboxylic acid (TCA) cycle in the matrix generates reducing equivalents NADH and FADH_2_. Electron transport chain (ETC) within the IMM uses NADH and FADH_2_ to create a proton gradient, enabling the production of ATP from adenosine diphosphate (ADP) by ATP synthase.

Structurally, mitochondria are double-membrane organelles with outer and inner membranes (OMM and IMM, respectively) (Figure 1). Embedded within the relatively smooth OMM are porins that permit the flow of molecules into the intermembrane space, the space between the OMM and IMM. This space contains various pro- and anti-apoptotic proteins, as well as proteins responsible for mitochondrial fusion and fission [12,13,14]. The IMM segregates the intermembrane space from the mitochondrial matrix and houses the protein complexes of the electron transport chain (ETC) and the ATP synthase [15]. The IMM forms multiple folds called cristae, which enlarge its surface area, thereby increasing mitochondrial capacity for OXPHOS [16]. Cristae structure can undergo dynamic rearrangements in response to various stimuli, and abnormal cristae morphology has been linked to various pathological conditions, including cancer [17]. The mitochondrial matrix contains enzymes involved in the tricarboxylic acid (TCA) cycle, which are the main producers of metabolites and reducing equivalents, such as nicotinamide adenine dinucleotide (NADH) and flavin adenine dinucleotide (FADH_2_) [15]. These reducing equivalents are then used by the ETC, creating an electrochemical proton gradient (mitochondrial membrane potential, ΔΨ_m_) that is necessary for OXPHOS and ATP production. Dysfunction in ETC components may result in electron leakage and oxidative stress, both of which have been implicated in various pathologies [18]. Similarly, changes in ΔΨ_m_ may also be indicative of changes in mitochondrial function and overall cellular health.

There is growing interest in methods to assess various aspects of mitochondrial structure and function in oncology applications, particularly in the context of metabolic reprogramming and changes in mitochondrial dynamics in chemotherapy-resistant cancer cells [19,20]. Moreover, cancer cells frequently exhibit altered mitochondrial function, even before therapy, resulting in enhanced invasiveness and metastatic potential [21,22]. This review focuses on the common techniques used to analyze mitochondrial structure and function, with a focus on their application in cancer research. Structural measurement approaches include optical and electron microscopy (EM)-based imaging to determine mitochondrial content, morphology, and subcellular localization in relation to other organelles, as indicators of potential dysfunction. Additionally, tagged proteins and transmission electron microscopy (TEM) can enable the visualization of mitochondrial dynamics (fission and fusion). Polymerase chain reaction (PCR)-based techniques can also be used for the quantification of mitochondrial deoxyribonucleic acid (mtDNA) copy number. Functional methods include the measurement of ROS production, ΔΨ_m_, calcium retention, and bioenergetic capacity, which provide information regarding mitochondrial health and metabolic status. In subsequent sections, the strengths and limitations of each method are reviewed and the scenarios in which these approaches can be used are presented, with a special focus on gynecologic malignancies. 

## 2. Structural Techniques and Parameters to Characterize Mitochondria Health

Structural changes that involve the ultrastructure of mitochondria, i.e., cristae morphology, mtDNA content or integrity, as well as mitochondrial dynamics, e.g., fission and fusion, are considered in this section. In cancer cells, mitochondrial dynamics include fusion, i.e., the merging of mitochondria, and fission, i.e., the splitting of mitochondria into separate units. Importantly, fission and fusion can occur simultaneously in a given cell, and the dynamics of these changes can contribute to cancer metastasis and drug resistance [23]. Additionally, cancer cells often display alterations in mtDNA copy number. Changes in mtDNA content, while structural in nature, are frequently linked with functional changes since mtDNA encodes for critical protein subunits of the ETC. Likewise, loss of mtDNA impacts cellular respiration/bioenergetics and the TCA cycle [24]. In this section, techniques to measure structural parameters, including microscopy and PCR-based methods, will be discussed, along with extracellular vesicle (EV) secretion and cargo. 

### 2.1. Optical Microscopy

Optical microscopy is a non-invasive method to quantitatively and qualitatively evaluate mitochondrial structure in both live or fixed cells [25]. Widefield fluorescence and confocal microscopy are often used to visualize mitochondria in cells with fluorescently-tagged resident mitochondrial proteins [25]. Alternatively, there are many commercially available fluorescent mitochondrial dyes such as MitoTracker™ (Invitrogen™, Thermo Fisher Scientific, Waltham, MA, USA) that specifically localize to mitochondria. Mitochondrial staining is often used in conjunction with other organelle trackers (such as LysoTracker™ (Invitrogen™)) to analyze the subcellular distribution of various imaging and therapeutic agents [26]. Moreover, some fluorescent dyes (such as JC-1) respond to changes in ΔΨ_m_ by changing their excitation and emission spectra, which can be visualized using optical microscopy [27]. Laser-scanning confocal microscopy can be used to obtain high-resolution 3-dimensional (3D) information from Z-stacks or conduct time-lapse imaging to visualize dynamic changes in mitochondrial morphology [25]. While conventional confocal microscopy allows for the visualization of mitochondria in living cells, it fails to provide information regarding the finer details of mitochondrial structure. The diameters of mitochondria are typically 250–500 nm, which approaches the diffraction limit of conventional light microscopes [28,29]. This necessitates more advanced approaches to examine mitochondrial structure and the distribution of proteins within mitochondria. 

**Figure 2:**
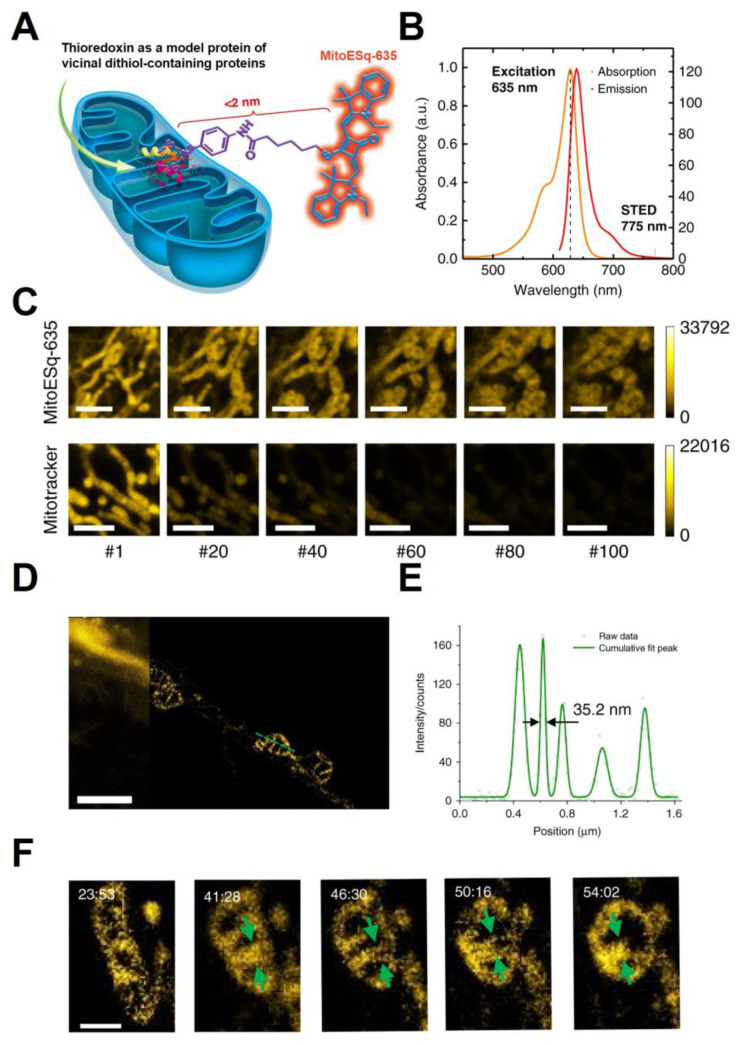
Quantitative STED nanoscopy with an enhanced squaraine variant dye. (**A**). MitoESq-635 specifically binds vicinal-dithiol-containing proteins in mitochondrial membranes. (**B**). Absorption and emission spectra of MitoESq-635. During STED, a 775-nm pulse laser can be used for depletion and a 635-nm pulse laser for dye excitation. (**C**). MitoESq-635 (0.1 μM, top panel) versus MitoTracker™ Deep Red (0.1 μM, bottom panel) photostability under STED imaging in unfixed HeLa cells. STED imaging parameters were equivalent for both dyes (excitation under 1.1 µW, 640 nm, STED beam of 30.2 mW average power at 775 nm, and 1 frame per s acquisition speed (imaging time: 0.66 s, recovery time: 0.34 s). Scale bar = 2.5 μm. (**D**). Confocal (left) and STED (right) imaging of mitochondria in HeLa cells. (**E**). STED nanoscopy using MitoESq-635 enables 35.2-nm resolution. (**F**). STED imaging of cristae transition from a line shape to a bubble shape (green arrows). Scale bars = 1 μm. Adapted with permission from Yang et al., Nature Communications; published by Nature Portfolio, 2020. Creative Commons License: http://creativecommons.org/licenses/by/4.0/ (accessed on 9 February 2023) [36].

Super-resolution techniques utilize the transition between the “on” and “off” states of a fluorophore to overcome the diffraction limit of conventional optical approaches [30,31,32]. Commercially available super-resolution techniques include stimulated emission depletion (STED), fluorescence photoactivation localization microscopy (FPALM) [33], and stochastic optical reconstruction microscopy (STORM) [34,35]. In STED microscopy, the singlet excited state of a fluorophore is reversibly silenced (depleted) at predefined positions of the diffraction-limited excitation regions. Since only the non-silenced fluorophores at a pre-defined position emit light, this allows objects to be distinguished with sub-diffraction resolution [36,37]. Dong et al. [38] utilized STED microscopy to investigate the horizontal transfer of red fluorescent protein (RFP)-labeled mitochondria from host cells to B16 mouse melanoma cells. The authors demonstrated that mtDNA-deficient cells acquired mtDNA from host cells by transferring whole mitochondria, resulting in the recovery of mitochondrial respiration and increased tumor formation. Current super-resolution techniques that allow the visualization of mitochondria in live cells are prone to photobleaching. Thus, new fluorophores are needed to achieve non-destructive time-lapse imaging at lower laser powers [39]. In a recent study, Yang et al. [36] developed an enhanced squaraine variant dye (MitoESq-635) to study dynamic changes in mitochondrial cristae morphology (Figure 2A,B). The designed conjugate could bind vicinal dithiol-containing proteins abundant in mitochondrial membranes, thereby labeling mitochondria with high efficiency as demonstrated by its co-localization with MitoTracker™ (Figure 2C). MitoESq-635 showed significantly enhanced photostability compared to MitoTracker™ Deep Red, enabling STED imaging in live HeLa cells over the course of 50 min at a resolution of 35.2 nm (Figure 2C–E). Time-lapse nanoscopy revealed thin tubular structures connecting mitochondria before and after fusion and fission that cannot be detected with traditional confocal microscopy. The authors also characterized width distributions of mitochondria and cristae at different stages of fusion and fission (Figure 2F). This technique enables strikingly detailed imaging of transient changes in cristae structure, which can be instrumental in studying mitochondrial response to various treatments and stressors. 

Another interesting direction is STED imaging in paraffin-fixed samples. Ilgen et al. [40] analyzed mitochondrial protein distributions in human rectal cancer tissues that had been stored up to 17 years. The authors demonstrated that routine clinical formalin fixation and paraffin embedding did not affect the distribution of proteins in the OMM. STED imaging revealed a distinct punctate localization of Tom20, an OMM-localized protein that recognizes and transports cytosolically synthesized mitochondrial preproteins, similar to that of cultured mammalian cells that were not subjected to paraffin embedding. Importantly, this approach enabled the visualization of Tom20 distribution and localization in mitochondria, distinct from other mitochondrial proteins, which was not possible with conventional confocal imaging. 

An evolving area is imaging of mitochondria in vivo. Optical imaging methods are generally limited by poor light tissue penetration, but using longer wavelengths helps avoid some of the endogenous absorption and minimize scattering. Two-photon excitation fluorescence (TPEF) microscopy is particularly suitable for in vivo imaging because it relies on the simultaneous absorption of two long-wavelength (typically near infrared) photons to excite a fluorophore [41,42,43]. Because two-photon absorption is a statistically rare process, TPEF is characterized by low background fluorescence and a high signal-to-noise ratio. Moreover, TPEF can be conducted in a time-resolved manner, measuring fluorescence lifetime, or the time that a fluorophore takes to return to its ground state post-excitation. Factors, such as the local pH, temperature, oxygenation, ion concentration, and enzyme activity, influence fluorescence lifetime. Thus, TPEF lifetime imaging can provide important functional information in cells and intact tissues. For example, TPEF imaging can detect endogenous fluorescence from two coenzymes, NADH and FAD, and changes in their lifetime can be used to non-invasively assess cell redox state and mitochondrial clustering [44]. This method has been applied to measure metabolic activity in tumor cells compared to normal cells [42,43,44,45,46,47,48]. Xylas et al. [43] identified unique features of mitochondrial organization that are reflective of the metabolic state. Specifically, the highly networked mitochondrial organization was associated with elevated glutamine consumption. Moreover, an automated imaging workflow based on this method was able to distinguish freshly excised normal and pre-cancerous human cervical tissues, indicating its potential diagnostic value. Heme precursor protoporphyrin IX (PpIX) is another endogenous fluorophore that can also be used to monitor bioenergetics in tumor cells compared to normal cells using TPEF [46]. Overall, TPEF- and lifetime-based optical imaging techniques hold promise to bridge the translational gap and enable the evaluation of mitochondrial state as part of the diagnosis and treatment planning for cancer patients. 

### 2.2. Electron Microscopy-Based Techniques

Mitochondria were among the first organelles characterized by EM because their size (0.5–3 µm) is close to the resolution limit for conventional light microscopy techniques. Early EM studies by Palade et al. [49] suggested that mitochondria were enclosed within two membranes: IMM and OMM. Textbook mitochondrial images reveal oval-shaped mitochondria in “orthodox” state, which is characterized by a relatively large matrix volume and a small space between the OMM and IMM [50]. More recent analysis of mitochondrial ultrastructure revealed that mitochondria exist either as interconnected (fused) tubular networks or as fragmented organelles [51,52]. At any given time, mitochondrial morphology is the result of a delicate equilibrium between fusion and fission, which can be altered by the cellular physiological state [3,53]. 

In cancer, mitochondrial ultrastructure is extremely heterogeneous. Early studies reported no significant differences between mitochondrial ultrastructure between healthy mouse mammary glands and mouse mammary adenocarcinoma [54]. Subsequent studies noted increased mitochondrial polymorphism and structural abnormalities in various types of tumors, including endometrioid and serous ovarian adenocarcinomas [55,56], squamous cell carcinomas [57], and others [58]. Recent evidence suggests that changes in cellular metabolic states, and subsequent alterations in mitochondrial ultrastructure in cancerous tissues can be indicative of drug resistance [8,59]. In a recent study, Signorile et al. [60] analyzed mitochondrial structure and function in human serous and mucinous ovarian cancer tissues using TEM. TEM imaging demonstrated that mitochondria in ovarian cancer cells were characterized by increased maximum length as well as decreased cristae width and junction diameter compared to the normal ovarian tissues. Furthermore, Ricci and co-authors [61] investigated alterations in mitochondrial structure in cisplatin-sensitive and cisplatin-resistant ovarian cancer patient xenografts. The authors found that platinum-resistant xenografts were characterized by a significantly lower number of mitochondria per μm^3^ than cisplatin-sensitive tumors. Moreover, platinum-resistant tumors more frequently displayed mitochondria with pathological morphologies, including mitochondria with abnormal shapes (e.g., concave or ring-like) or with evidence of cytolysis. The increased frequency of mitochondrial abnormalities was accompanied by an increase in oxidative metabolism, reported in a separate study [62]. It is increasingly evident that mitochondrial abnormalities in cancer are often reflected in altered ultrastructures. However, the nature of these ultrastructural changes can vary depending on the tumor type. 

Although EM can provide important 2-dimensional (2D) information on mitochondrial morphology, heavy sample processing is required, and real-time data are lacking. 3D visualization techniques offer a more comprehensive picture of mitochondrial structure. One such technique is electron tomography [63,64]. To obtain 3D images, a specimen is tilted at small regular intervals (1–2°), and a series of images (typically consisting of 60–120 images) is obtained similar to TEM [65]. These images are then aligned to the common origin, and a 3D map can be calculated at 2–6 nm resolution, providing enhanced details often not visible in 2D images. For example, electron tomography revealed that cristae are interconnected through a network of ~28 nm tubular junctions [66,67,68]. Additionally, electron tomography was used in conjunction with fluorescence microscopy to investigate structural changes that occurred during etoposide-induced apoptosis in HeLa cells [69]. The authors observed IMM remodeling into many separate vesicular matrix compartments, followed by protein release. 

**Figure 3:**
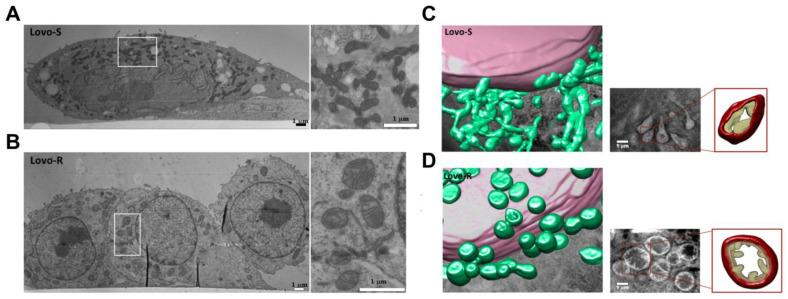
EM and cryo-SXT imaging of mitochondria in doxorubicin sensitive (LoVo-S) and doxorubicin-resistant (LoVo-R) colon carcinoma cells. (**A**). LoVo-S exhibit flattened morphology and strong adherence to a cell culture dish. LoVo-S mitochondria have well-preserved ultrastructure and electron-dense matrix (**B**). LoVo-R cells are more rounded and appear to have a weak relationship with the substrate. LoVo-R mitochondria are characterized by a pale matrix, disarrangement of the cristae and moderate cristolysis. (**C**). Cryo-SXT ultrastructural analysis of mitochondria reveals notable differences between LoVo-S and (**D**). LoVo-R mitochondrial morphology. Images represent three-dimensional reconstructions obtained by manual segmentation of the surface boundaries, wherein the nucleus is depicted in violet and mitochondria in green. The insets in (**C**,**D**) represent one slice of the tomogram. Scale bars = 1 μm. Adapted with permission under a Creative Commons Attribution (CC BY) license from Moscheni et al., Cancers; published by MDPI, Basel, Switzerland, 2019 [70].

Soft X-ray Cryo-Tomography (cryo-SXT) is another emerging imaging technique that provides 3D maps of vitrified whole-cell samples at nanometer-resolution. Importantly, cryo-SXT allows the visualization of whole cells and their structural components without staining or sectioning, keeping them as close as possible to their native state. Moscheni et al. [70] investigated 3D mitochondrial ultrastructure in doxorubicin-sensitive and -resistant human colon carcinoma cells (Figure 3). Doxorubicin is known to induce alterations in mitochondrial structure and function, which may be one of the mechanisms by which it promotes multi-drug resistance. The authors applied cryo-SXT to create 3D renderings of doxorubicin-sensitive and doxorubicin-resistant cancer cells at nanometer resolution. EM revealed that doxorubicin-sensitive cells were characterized by irregularly shaped and sized mitochondria with dark condensed matrix and irregular cristae, as opposed to more regularly sized oval-shaped mitochondria with electron-lucid matrix found in doxorubicin-resistant cells (Figure 3A,B). Further, the 3D nano-rendering of whole cells revealed that mitochondria in doxorubicin-sensitive cells were elongated, while those in doxorubicin-resistant cells were close to spherical (Figure 3C,D). Interestingly, mitochondria in doxorubicin-sensitive cells appeared as an interconnected tubular network, while those in doxorubicin-resistant cells resembled a fragmented state. Functionally, a highly fragmented mitochondrial state in drug-resistant cells was characterized by increased spare respiratory capacity. This study illustrates how 3D rendering of representative volume regions can provide important structural information regarding mitochondrial organization, which reflects cellular metabolic fitness and drug sensitivity. 

Another technique that can provide both structural and compositional information at the nanoscale is focused ion beam scanning electron microscopy (FIB-SEM). FIB-SEM, which involves making precise cuts with a focused ion beam and imaging the exposed surface, has recently become available for biological sample analysis [71,72,73,74]. Most FIB instruments use liquid metal ions, such as liquified Gallium, channeled through a thin needle. Voltage application results in the emission of metal ions, which can be directed towards the sample. The stream of heavy Gallium nuclei causes sample milling at a rate that is proportional to substrate properties. Typical sample processing involves fixation or freezing [75], staining with heavy metals, and resin embedding [73,76]. Recently, Murphy et al. [77] applied FIB-SEM to investigate mitochondria in murine methylmalonic acidemia. Methylmalonic acidemia is an autosomal recessive genetic disorder, which is manifested as a malfunction of an enzyme converting methylmalonyl-CoA to succinyl-CoA and the formation of enlarged mitochondria. FIB-SEM imaging of mouse liver cells revealed a larger mitochondrial surface-to volume ratio, more convoluted morphology, and a broader size distribution in cells from the diseased animals compared to healthy controls. Importantly, this study demonstrated the feasibility of using FIB-SEM as a quantitative tool to characterize subcellular 3D changes under pathological conditions. 

In summary, EM-based techniques enable 2D and 3D analysis of mitochondrial structure at the nanoscale. Table 1 provides a summary of the strengths and limitation of the various types of microscopies discussed in the context of mitochondrial status, with a focus on disease. While these microscopy-based methods provide insights into mitochondrial organization, they should be combined with functional measurements for a more complete evaluation of mitochondrial state.

### 2.3. Evaluation of mtDNA Content or Integrity Using PCR

The depletion of mtDNA has been associated with deficiencies in the ETC and pathologies associated with altered mitochondrial function [86], such as type II diabetes, neurodegenerative disorders, and even cancer in humans [87,88,89,90]. Examining cellular mtDNA content can, therefore, provide insight into the state of metabolic function in a cell. To isolate DNA from cells or tissue samples, kits, including the QIAamp^®^ DNA Mini Kit (QIAGEN, Hilden, Germany) or DNeasy^®^ Blood and Tissue Kits (QIAGEN, Hilden, Germany), can be used [87,91]. The amount and/or integrity of mtDNA after DNA isolation can be measured using a variety of methods, including gel electrophoresis and Southern blot, high-performance liquid chromatography (HPLC), or quantitative real-time polymerase chain reaction (qPCR) [86,92,93,94,95]. One advantage of using PCR compared to gel electrophoresis coupled with Southern blot for the analysis of mtDNA integrity is that the amount of DNA needed for PCR is significantly smaller, in the nanogram range, than that required for Southern blotting, which is in the microgram range [86,93,94]. qPCR is also advantageous because mtDNA isolation, which artificially oxidizes the genome posing a challenge for interpretation of mtDNA integrity analysis, is not required [93,96,97]. As a result, qPCR is the method of choice to determine cellular mtDNA content. When using this technique, it is important to note that primers/probes for both the mitochondrial genome and nuclear genome, specifically a single copy nuclear gene, must be used for normalization, since copy numbers can differ between nuclear and mitochondrial genomes in the order of 4000-fold [86,98]. It is also critical that primers are specific to the target fragment, including from the mtDNA since mitochondria-like sequences are present in the nuclear genome. Another consideration for mtDNA analysis, when working with tissue, is to include an age- or tissue-matched control for comparison, since mtDNA content changes with age and is highly dependent on the tissue type being examined [86,99,100,101,102]. 

In the context of cancer, studies have reported that decreased mtDNA content, examined by qPCR, is associated with ovarian cancer progression or severity. Specifically, Wang et al. [103] reported that while mtDNA content in ovarian tumor cells was significantly higher than in normal ovarian tissue, mtDNA copy number decreased in high-grade carcinomas compared to low-grade carcinomas, indicating that mtDNA content does not necessarily change linearly with disease progression and may be of diagnostic value. Other studies have reported that compared to benign tissue, mtDNA levels in ovarian tumor tissue, measured via qPCR, did not significantly differ, although tumor tissues had much higher mitochondrial content and respiratory chain activities than normal tissue [104]. While no significant difference in mtDNA content was observed in benign versus tumor tissues, the authors noted that mtDNA content was significantly decreased in stage IV tumors by an average of ~28% compared to stage I [104].

Importantly, cisplatin, a platinum-based chemotherapy agent frequently used to manage gynecologic malignancies, forms adducts with mtDNA and impairs the transcription of ETC proteins, thereby increasing mitochondria-induced ROS and triggering cell death [105,106,107]. Indeed, impaired response to chemotherapy, including to cisplatin, has been observed in mtDNA-depleted cells relative to cells that contain mtDNA, further demonstrating the importance of mtDNA in cisplatin-mediated toxicity [108,109,110]. Although mtDNA is a target of cisplatin, mtDNA copy number changes have been implicated in resistance to chemotherapy. For example, a study by Mei et al. [111] found that lowering mtDNA copy number sensitized the cells to cisplatin while increased mtDNA content enhanced tumor survival by preventing apoptosis. Therefore, reducing, but not fully depleting, mtDNA content in tumor cells may be a strategy to target platinum-resistant cells [110,111]. When measuring mtDNA content, an important follow-up experiment involves examining mitochondrial copy number, using Western blotting to probe for mitochondrial proteins, such as voltage-dependent anion-selective channel (VDAC), transcription factor A mitochondrial (TFAM), cytochrome B (CYTB), or NADH dehydrogenase subunit 1 (ND1), and normalizing levels to a nuclear target, such as RNA polymerase II. 

Digital PCR (dPCR) is a valuable technique not only to quantify mtDNA content, but also to detect genetic alterations, including single nucleotide polymorphisms (SNPs), copy number variation, genomic rearrangements, and gene quantification [112,113,114,115,116]. dPCR is comparable to qPCR in that it uses the same reagents and workflow. However, in dPCR, the samples are fractionated into thousands of nanoliter partitioned reactions. Ideally, these partitioned reactions contain no, or only a few copies of the target sequence. PCR amplification of the target sequence occurs in each micro-reaction, which reduces PCR efficiency bias (qPCR is PCR efficiency-dependent) and the presence of potential PCR inhibitors. Poisson’s distribution is applied to quantify the number of positive partitions over negative partitions. This gives dPCR the advantage of quantification in copies per microliter without the need for a standard curve or reference gene normalization. Currently, there are two mainstream partitioning technologies, namely water-oil emulsion droplet technology, called droplet digital PCR (ddPCR), and microfluidic chamber technology. Both technologies fractionate the sample into 20,000 partitions and produce greater precision and improved reproducibility over traditional qPCR [117]. In addition, dPCR is highly sensitive at distinguishing mutations in DNA (differing by one nucleotide) with a detection limit of 0.001% [118].

Like qPCR, dPCR can be used to explore mtDNA copy number variation in cancer cells but with increased efficiency [117,119,120,121,122]. Understanding mtDNA copy number in cancer cells is critical since alterations have been associated with invasiveness and patient survival [120,123,124]. For example, a group in Sweden recently performed a prospective study reporting that mtDNA copy number was significantly associated with the incidence and mortality of various cancer types [122]. Specifically, mtDNA copy number was significantly higher in the blood of breast cancer patients compared to cancer-free patients. Additionally, although lower mtDNA copy number was associated with decreased reproductive cancer risk, it was associated with increased risk of mortality resulting from reproductive cancer [122]. Other studies have found that increased mtDNA copy number in blood of suspected cancer patients was associated with decreased risk of solid tumors [117]. ddPCR has also been used to evaluate how mtDNA copy number changes in human colonic epithelial cells mimicking colorectal cancer initiation and progression. In this study, O’Hara et al. [120] found that mtDNA copy number decreased in cells overexpressing KRAS or KRAS and MYC [125,126]. A more recent area of exploration is using ddPCR to detect mtDNA mutations or deletions [121], which may prove useful in the context of cancer since biomarkers are lacking. Table 2 summarizes the strengths and limitations of these PCR-based approaches.

### 2.4. Extracellular Vesicle (EV) Secretion and Cargo

The term EV encompasses exosomes, microvesicles, and other membrane-bound particles secreted by cells [129]. Generally, EVs carry an internal and external cargo of proteins, DNA (both genomic and mitochondrial) as well as other nucleic acids (e.g., messenger ribonucleic acids (mRNAs), microRNAs (miRNAs), and long non-coding (lncRNAs)), and intact organelles such as mitochondria [130]. As a result, they have emerged as a new class of potential clinical biomarkers for personalized medicine interventions. EVs have been acknowledged as crucial agents of communication between cells, both in healthy and disease states. Proteins and RNAs have been the most extensively researched components, and the role of EVs in mediating communication between cells is now understood to be much more complex. Upon their uptake by cells, EVs have significant modulation capability for both intracellular and intercellular signaling, resulting in altered disease pathology and cell responses to external stimuli in recipient cells [131]. Thus, studying EVs and their cargo can be highly beneficial for cancer patients, from monitoring disease progression to predicting treatment outcomes. 

EVs comprise a heterogeneous population of membranous vesicles that are generated via diverse mechanisms, but the two main EV subpopulations include ectosomes (100–1000 nm in diameter) and exosomes (50–150 nm in diameter). Ectosomes comprise a highly heterogenous type (both in composition and size) of EVs, such as oncosomes and microvesicles, that are generated at the plasma membrane from its outward budding. Exosomes, on the other hand, are produced inside the cells from multivesicular bodies by inward budding of the endosomal membrane, which results in the formation of very small vesicular structures contained within the endosome lumen and are released as part of exocytotic processes [132]. These extracellular components can modulate physiological processes and can be noninvasively accessed from biofluids, such as blood plasma, urine, or saliva, to serve as molecular biomarkers of both normal and pathophysiological cellular processes, including early cancer detection [133,134,135,136,137,138,139,140]. Recently, a new class of EVs has been identified as exomeres, with a size range smaller than 50 nm and a mean diameter of 35 nm. Interestingly, one study [141] showed that there was specific enrichment of membrane-associated proteins in exosomes, which were relatively depleted in exomeres. However, proteins associated with mitochondria were found to be packaged in exomeres. 

One of the most frequently used methods to isolate EVs is ultracentrifugation. During this process, serum, cells, or other biofluids are spun down at high centrifugal force and EVs that travel the length of the tube are pelleted at the bottom. Those EV pellets can be resuspended and used for downstream analyses, which may include nanoparticle tracking analysis (NTA), Western blotting, or miRNA quantification [142]. Other techniques to isolate EVs include density gradient centrifugation, size exclusion chromatography, microfluidics [143], size-based filtration [144], and polymer-based precipitation. As with most techniques, each method of separation and purification comes with a set of advantages and disadvantages, in this case primarily concerning EV yield and the presence of non-EVs [145]. Although studies have shown that multiple centrifugation steps decrease the overall EV number due to damaged EVs, this process also decreases the amount of non-EVs present in samples, which is a clear advantage and consideration when selecting a separation method [142].

It is important to note that a standard EV isolation protocol is lacking [146,147]. This makes it challenging to compare studies using differing isolation methods due to variations in EV purity, quality, and quantity. While ultracentrifugation is considered the “gold standard” for EV isolation, this technique has limitations. For example, ultracentrifugation may lead to vesicle aggregation and the efficiency of generating EV pellets may differ depending on centrifuge rotor [146,148,149].

To date, the most investigated EV-related markers have been miRNAs, which play crucial roles in the regulation of gene expression. In the context of cancer, dysregulated miRNAs can enhance proliferative signalling and metastatic capacity, induce angiogenesis, and promote evasion of therapeutic response, making them valuable biomarkers [150,151]. For example, in prostate cancer, miR-375 has been shown to correlate with diagnosis as well as a worse prognosis via its ability to induce resistance to docetaxel, potentially by targeting SEC23A and YAP1 [152,153,154]. In ovarian cancer, a systematic review found that miR-223, miR-1246, miR-433, miR-21, and miR-1307 were potential biomarkers with diagnostic value as they have been shown to promote cell resistance to paclitaxel by various pathways, including PTEN-PI3K/AKT [131,155,156,157,158,159]. 

While differentially expressed proteins and RNAs have received considerable attention, the discovery of cancer-derived EVs containing DNA offers new possibilities for studying cell-to-cell communication and monitoring diseases. Cell-free DNA (cfDNA) is seen as the definitive molecular biomarker in liquid biopsy. However, challenges associated with using cfDNA include the need for large sample volumes and sample instability [160]. By contrast, cfDNA associated with EVs has recently garnered intense interest as a potential untapped reservoir in DNA-based liquid biopsy. Intact vesicles seem to protect the DNA in circulation and during the isolation steps, making this another attractive advantage of EVs [133]. However, cfDNA is derived from cells that are either undergoing apoptosis or necrosis, which may not accurately represent the overall population of viable cells within the tumor [161].

DNA contained within EVs (EV-DNA), in addition to cfDNA, has been implicated in physiological processes in various diseases as a potentially effective biomarker [162,163]. Importantly, EV-DNA is released by cells that are still metabolically active and mirrors the genetic changes present in the cancer cells from which they originate [164,165]. EV-DNA can be genomic (ssDNA [166], dsDNA [164,165]) or mitochondrial (mtDNA [167]) in origin, and this distinction can influence functionality. Further, the location of the EV-DNA (on the EV surface or internal in the EV lumen) may have importance in cellular behavior and homeostasis [168]. The unique composition and packaging of EV-DNA suggest that it is intended for use beyond its originating cell, raising questions about the mechanisms involved in its transport and functions outside the cell. 

While there is still much to be discovered about these processes, some theories have emerged regarding the origin and loading of DNA, or mtDNA, into EVs [169]. It has been postulated that EV-DNA can originate from cytoplasmic DNA fragments that resulted from either regular DNA metabolism or DNA damage [170]. Dead cells releasing cfDNA may also contribute to the presence of EV-DNA by adhering to the surface of EVs that are released [171]. Pertaining to the presence of mtDNA in EVs [167,172], one study showed that mitochondria-derived vesicles generated from oxidative damage could be targeted to endosomal pathways, where their DNA contents are packaged into exosomes [173]. Lysosomal vesicles, which are rich in mitochondrial content due to mitochondrial autophagy, may be released as EVs [174]. Regarding loading of DNA into EVs, studies suggest that this may occur within the cytoplasm through the use of endosomal tetraspanins such as CD63 [175]. Nevertheless, further studies are necessary to elucidate the ways in which both genomic DNA and mtDNA are loaded into and released via EVs. 

Moreover, the purpose and mechanisms of EV-DNA driven communication between cells, particularly in cancer biology, remain largely unknown [169]. One study proposed DNA secretion via EVs as an integral mechanism in clearing cytosolic DNA to maintain cellular homeostasis and avoid senescence and apoptosis [176]. Another study showed that in cancer, EV-mtDNA act as an “infectious” mediator (i.e., horizontal transfer) of therapy resistance in OXPHOS-dependent human breast cancers, leading to metastatic progression [172]. 

EV-DNA has proven to be a useful tool in identifying mutations linked to cancer cells and tumors. Despite promising results [162,177,178], the clinical application of EV-DNA for cancer diagnosis is yet to be fully validated. Only several studies registered on ClinicalTrials.gov have incorporated EV-DNA as a cancer biomarker, including those for soft tissue sarcoma [179], colorectal cancer [180], and non-small cell lung cancer (NSCLC) [181,182]. Many previous studies using EV-DNA to detect cancer-related mutations face the challenge of ensuring EV purity [183]. A recent study [184] confirmed the contamination of cell culture EV samples isolated using ultracentrifugation with naked DNA (i.e., non-EV DNA). They also showed the composition of DNA-positive EVs largely varies, ranging from 30% to 80% depending on the cell type. Enriching only DNA-containing EVs will enhance the sensitivity of cancer mutational analyses, enabling the detection of microscopic clones or even the earliest stages of cancer relapse or metastasis.

To fully comprehend the roles of EVs in tumorigenesis and tumor evolution, it is vital to understand the mechanisms and conditions under which EVs are shed and the content they carry. Understanding the kinetics of EV-DNA packaging and EV release patterns during a single cell cycle, as well as factors that affect this process, is essential. Once a more comprehensive understanding of these processes is achieved, EVs, which are present in almost all body fluids, and the stability of EV-DNA, compared to cfDNA, due to its protection by the lipid bilayer, can be leveraged. This knowledge could enable the development of a characterization system for EV-DNA with specific mutations, potentially enabling a robust system for liquid biopsies.

## 3. Functional Parameters to Understand Mitochondrial Health

A variety of factors can lead to the modulation of mitochondrial function. Improvements often lead to enhanced mitochondrial health while impairments lead to mitochondrial dysfunction and can induce cell death. In cancer, such alterations in mitochondrial function can determine cell invasiveness, metastasis, and response to therapy. Therefore, measuring mitochondrial parameters in cancer cells is critical to understanding mitochondrial processes underlying tumor progression and for the development of targeted therapies. While functional and structural measurements of mitochondrial health are often interrelated, functional parameters, including ROS production, calcium retention, ΔΨ_m_, and bioenergetic capacity, can provide insight into oxidative stress status, homeostasis, membrane integrity, and metabolic capabilities. In this section, optical and non-optical approaches to quantify these parameters, as well as other common techniques, will be discussed. 

### 3.1. Reactive Oxygen Species (ROS) Production 

The term “ROS” encompasses a group of highly reactive molecules including free radicals like superoxide and nitric oxide, and non-radicals like hydrogen peroxide and singlet oxygen [185]. ROS production can provide insights into the overall health of a cell, as imbalances, notably excess ROS accumulation, can lead to oxidative stress and cellular damage. Cancer cells often demonstrate elevated rates of ROS production that are correlated with tumor development, progression, and metastasis [185]. Elevations in ROS production in cancer cells can be due to increased OXPHOS, cytokine activation, or upregulated oncogenic signaling pathways such as MAPK/ERK and PI3K/AKT [185]. Hypoxic tumor microenvironments may also stimulate ROS generation [186]. To maintain homeostasis and control oxidative stress, specific ROS-related enzymes are present in the cell including superoxide dismutase and glutathione peroxidase. Depending on the ROS of interest, specific free radical and non-radical species can be measured alongside ROS-specific enzymes using fluorescence-based assays or OxyBlots [187].

Fluorescent dyes are often used to measure ROS production in live cells but should be carefully selected depending on which species is of interest. For example, superoxide can be measured using the MitoSOX™ (Invitrogen™) dye while Amplex^®^ Red (Invitrogen™) reagent can be used to detect hydrogen peroxide. A variety of genetic probes are available to quantify hydrogen peroxide production, including HyPer, HyPer2, and HyPer3 [185]. When using these dyes or probes, it is important to include controls, such as a mitochondrial uncoupler or a pH-insensitive form, to ensure that the sources of the signals are mitochondria and ROS, respectively. Numerous studies have used fluorescent ROS dyes to understand how oxidative stress affects tumorigenic potential [188,189,190,191]. 

In addition to fluorescent dyes, oxidative damage to proteins can be determined using an OxyBlot as an indicator of an increase in steady state levels of ROS. An OxyBlot detects carbonyl groups added to protein side chains by oxygen free radicals or other reactive species to characterize protein oxidation. The reactive species can be generated by numerous endogenous and exogenous sources including cellular metabolism and environmental contaminant exposure, respectively [192,193,194]. Often, oxidative species preferentially attach to specific protein side chains, such as methionine, histidine, and tyrosine, to form cysteine disulfide bonds [195,196,197,198]. Measuring and understanding protein oxidation during disease progression is critical. Protein modification can alter enzymatic activity [199,200,201] and transcription [202,203,204], and oxygen-derived free radicals play key roles in the development of human disease, including cancer [205]. Immunodetection of carbonyl groups present in protein side chains using an OxyBlot involves derivatization of carbonyl groups on proteins using dinitrophenyl hydrazine followed by polyacrylamide gel electrophoresis and Western blotting to visualize total protein carbonyl levels. Cell lysates, e.g., prepared using RIPA buffer followed by centrifugation, are a common starting material for this technique, but purified proteins can also be used. In the context of cancer, increased levels of protein carbonyls, measured via OxyBlot, have been linked to disease aggressiveness and invasiveness in serous ovarian carcinoma. In a study by Mehrabi et al. [206], serous ovarian carcinoma tissues, compared to normal ovarian tissue, displayed increased levels of protein carbonyls, which further increased with advanced disease stage, indicating that oxidative damage may influence ovarian cancer progression. 

### 3.2. Mitochondrial Membrane Potential (ΔΨ_m_)

ATP production by OXPHOS is one of the vital cellular functions of mitochondria [207,208]. During OXPHOS, protons are pumped from the inner- to the inter-membrane space of a mitochondrion, and an electrochemical gradient is created across the IMM through the ETC. The protons then flow back into the mitochondrial matrix, which provides the energy for phosphorylation of ADP to produce ATP. The force that mediates proton flow into the mitochondrial matrix is called the proton motive force and is driven by a pH and a negative electrical gradient, also known as ΔΨ_m_ [208,209]. 

The ΔΨ_m_ component of the proton motive force of live cell mitochondria can be characterized using cationic fluorescent dyes, such as methyl and ethyl ester of tetramethylrhodamine (TMRM and TMRE), Rhod123 (Rhodamine 123), and JC-1 (5,5′,6,6′-tetrachloro-1,1′,3,3′- tetraethylbenzimidazolylcarbocyanine iodide) [210,211,212]. Positively charged fluorescent dyes accumulate in mitochondria in a manner that is proportionate to the negative electrical gradient, i.e., ΔΨ_m_. The fluorescence signal from mitochondria is then detected by optical methods, including flow cytometry, microplate spectrophotometry, and fluorescence microscopy. One key consideration when examining ΔΨ_m_ via fluorescent dyes, such as TMRE or TMRM, is whether the dyes are used in quenching or non-quenching mode [213,214]. In quenching mode, the dye is loaded at relatively high concentrations, so that the dye molecules form quenched aggregates inside the mitochondria. The dye molecules are subsequently unquenched and become detectable when they are released from mitochondria due to a decrease in ΔΨ_m_, e.g., in response to treatment. Quenching mode is preferred for real-time monitoring of an acute effect of an experimental intervention on ΔΨ_m_. Conversely, in the non-quenching mode, the dye molecules are used at lower concentrations. In this case, the mitochondrial uptake of the unquenched fluorescent probes is proportional to ΔΨ_m_. Non-quenching mode is usually preferred when chronic effects of an experimental treatment on ΔΨ_m_ are being evaluated [213,214,215,216]. Quenching vs. non-quenching considerations do not apply, however, to the JC-1 assay, since dye aggregation leads to a spectral shift from green to red fluorescence emission [216]. An important consideration for the JC-1, and even Rhod123, dye, is loading concentration, since high concentrations can lead to dye accumulation in non-mitochondrial organelles. Additionally, fluorescence measurements should be normalized to that of cells treated with carbonyl cyanide m-chlorophenyl hydrazone (CCCP) or carbonyl cyanide-p-trifluoromethoxyphenylhydrazone (FCCP), which are mitochondrial uncouplers.

In cancer cells, ΔΨ_m_ is often elevated compared to normal cells [217,218,219]. This hyperpolarization can be indicative of enhanced mitochondrial capacities compared to normal cells, where ΔΨ_m_ is lower [220]. Additionally, increased ΔΨ_m_ can be a proxy for malignancy [219]. Although changes in ΔΨ_m_ can be transient and potentially reversible, studies have reported incidences of increased ΔΨ_m_ in cancer cells and therapy-resistant cancer cells [20,221,222]. Specifically, a study by Grieco et al. [221] showed that ovarian cancer cell mitochondrial phenotypes were characterized by disorganized cristae and increased ΔΨ_m_. Additionally, a study by Rickard et al. [20] reported that in ovarian cancer cells exposed to perfluoroalkyl substances (PFAS), which are prominent environmental contaminants, ΔΨ_m_ increased in chemotherapy-responsive and -resistant groups. Importantly, decreasing ΔΨ_m_ or inducing depolarization can enhance the susceptibility of tumor cells to treatment, or can shift a cell towards apoptosis [223,224,225,226,227]. One treatment approach that has been shown to decrease ΔΨ_m_ is photodynamic priming (PDP). PDP is a light-based, sub-cytotoxic treatment modality that can enhance tumor cell susceptibility to conventional therapies through the production of reactive molecular species [228]. In a study by Rickard et al. [229], PFAS-exposed ovarian cancer cells that had elevated ΔΨ_m_ and were chemotherapy-resistant demonstrated decreased ΔΨ_m_ after PDP and were re-sensitized to chemotherapy. Decreased ΔΨ_m_ after PDP has also been reported by others [230,231,232,233]. 

It is important to note that while ΔΨ_m_ can be useful for understanding mitochondrial and cellular health, there are several disadvantages inherent to specific dyes that must be considered prior to evaluating this parameter [27,216]. These include slow permeabilization times and the potential for photobleaching [216]. It is also noteworthy that the fluorescence intensity provided by the above-mentioned dyes can also be affected by the number of mitochondria in the population. Thus, it is indicated that other measurements of mitochondrial content be included when using dyes to determine the ΔΨ_m_, particularly when data are acquired by techniques other than microscopy. 

### 3.3. Calcium Retention Capacity

Calcium plays a key role in homeostasis, however, in the context of mitochondria, calcium signaling is critical for apoptosis. In fact, dysregulation of calcium signaling can induce cell death [234,235]. Under normal physiologic conditions, increases in mitochondrial calcium levels lead to increases in bioenergetics, specifically ATP production [234,236,237,238]. In some cases, when calcium levels increase too quickly or persistently, mitochondria become overloaded, which can cause membrane swelling or permeabilization and even apoptosis [234,236,239,240,241,242,243,244]. It is important to note that calcium uptake in mitochondria only occurs over a certain concentration range [245], and thus may or may not have an impact on cytosolic calcium signaling. Often, calcium levels are measured alongside ΔΨ_m_ and apoptotic markers, e.g., cytochrome c release, caspase activity, and nuclear fragmentation, to better understand changes in mitochondrial membrane permeability [246]. 

Similar to ΔΨ_m_, cytosolic and mitochondrial calcium levels can be measured using different fluorophores. For example, cytosolic calcium levels can be evaluated using Fura2, Fura2FF, Fluo3, and Fluo3FF while mitochondrial calcium levels can be measured using Rhod2 and Rhod2FF [234]. Other calcium-sensitive fluorescence dyes that can be used include Oregon Green 488 BAPTA-1, Calcium Green-5N, and Fluo-5N [236]. Once cells or mitochondria are labeled with fluorophores, fluorescence can be measured using fluorimeters or fluorescence imaging [234,236,246]. Similar to other techniques in this review, permeabilized cells or isolated mitochondria can be used to determine calcium retention capacity. Cell permeabilization can be performed using agents such as digitonin or saponin. Mitochondrial isolation is more complex, as it relies on 2-step centrifugation to remove intact cells and cell debris before separation of mitochondria from other organelles [247]. The use of isolated mitochondria tends to have a few more disadvantages, since the isolation process can lead to damage or loss of mitochondria [236,248,249,250].

Calcium dysregulation can lead to a variety of diseases, including cancer [251,252,253,254]. In cancer, aberrant calcium signaling can enhance cell proliferation and angiogenesis [254,255]. Importantly, disruptions in calcium homeostasis are associated with increased apoptosis and autophagy in cancer cells [256,257], indicating that calcium dysregulation can produce effects on both ends of the spectrum. In ovarian cancer, calcium may play a key role in proliferation [258,259], which is critical as certain reproductive hormones induce changes in cellular calcium levels [254,260,261]. 

### 3.4. Mitochondrial Bioenergetics: Glycolytic and Respiratory Capacity Measurements

Since studies have reported that tumor cells often have increased bioenergetic capacities compared to healthy cells [19,262,263,264,265,266,267], understanding how the bioenergetic profiles of tumor cells can be exploited for therapeutic benefit may be important for improving survival outcomes. One main method of measuring glycolytic and respiratory capacities of cells involves using a Seahorse Extracellular Flux Analyzer (Agilent, Santa Clara, CA, USA) [268,269,270,271].

The Seahorse Extracellular Flux Analyzer measures cellular capacity for glycolysis and OXPHOS in real-time by analyzing extracellular acidification rate (ECAR) and oxygen consumption rate (OCR), respectively [269]. Prior to measuring cellular bioenergetics using this platform, several optimization steps must be performed, including determining optimal cell density as well as the optimal concentrations of each injection compound, which include glucose, oligomycin, 2-deoxyglucose, FCCP, and rotenone/antimycin A [269]. To measure ECAR, injections of glucose, oligomycin, and 2-deoxyglucose are critical because they stimulate glycolysis, shift the cell towards glycolysis by blocking ATP synthase, and inhibit glycolysis, respectively [270]. To measure OCR, injections of oligomycin, FCCP, and rotenone/antimycin A are critical because they shift the cell towards glycolysis by blocking ATP synthase, increase proton permeability of the IMM to stimulate ETC activity, and inhibit complexes I and III of the ETC, respectively [270]. 

Upon determination of the optimal cell density and concentration of the injection compounds, the assay can be run to determine real-time ECAR and OCR. Basal ECAR is measured prior to injection of glucose, which should increase ECAR; oligomycin, which should further increase ECAR; and 2-deoxyglucose, which should decrease ECAR [19]. Basal OCR is measured prior to injection of oligomycin, which should decrease OCR; FCCP, which should increase OCR; and rotenone/antimycin A, which should decrease OCR [269,271]. Differences between ECARs and OCRs after each injection should be compared between tumor cells and healthy cells to better understand how their energy profiles differ post-injection of various ETC substrates or inhibitors. However, it is critical to normalize data per well using cell number or protein content. Importantly, substrate preference can also be analyzed. For example, cellular preferences for using glutamine, glucose, or fatty acids to preferentially fuel the TCA cycle can be determined. Metabolomic analysis can further reveal differences in TCA cycle metabolites in normal vs. cancer cells and may be critical for understanding the development and treatment of pathologic conditions [272,273,274]. 

In the context of cancer, a study by Dier et al. [262] examined the bioenergetic profiles of ovarian clear cell carcinoma (OCCC) cell lines in comparison to other epithelial ovarian carcinoma subtypes. The authors reported that ES-2 and TOV-21-G cells, which are thought to be representative of OCCC, were highly metabolically active, since their OCR and ECAR were elevated compared to normal ovarian surface epithelial cells. Interestingly, OCR readings between OCCC cells and those of other ovarian cancer subtypes did not significantly differ, suggesting that mitochondrial oxygen consumption may be comparable in ovarian cancer cells across subtypes. Dier et al. [262] also reported that cells with high OCRs and ECARs were better able to form spheroids, although no clear association between bioenergetic profile and response to chemotherapy was observed. 

**Figure 4:**
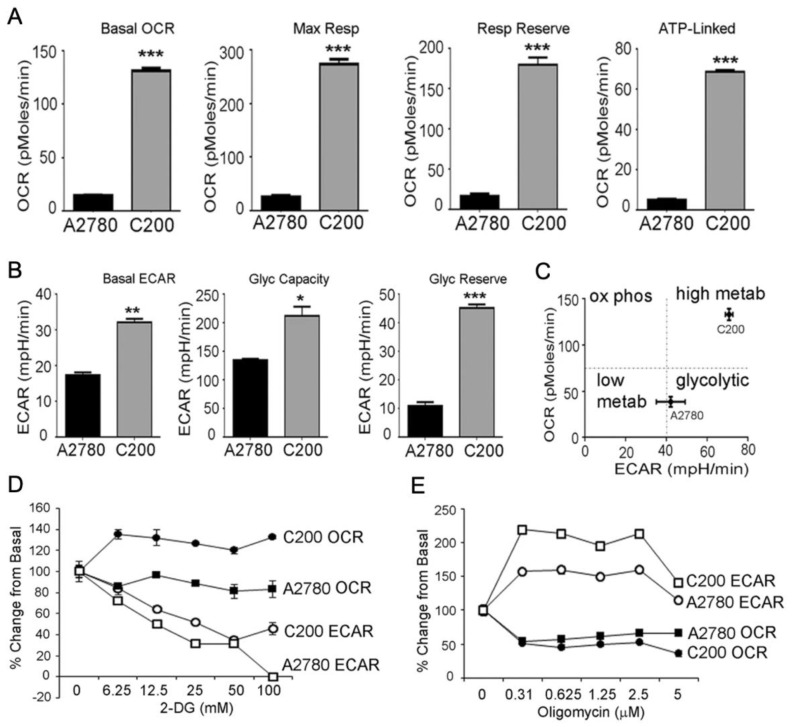
Quantifying bioenergetic alterations in chemoresistant and chemosensitive ovarian cancer cells. (**A**) Compared to chemosensitive A2780 cells, chemoresistant C200 cells displayed increased basal OCR, maximum respiratory capacity, and respiratory reserve, which indicates higher baseline mitochondrial respiration and enhanced capacity to use mitochondria for energy production. (**B**) Basal ECAR, glycolytic capacity, and glycolytic reserve are higher in C200 cells than A2780 cells, suggesting chemoresistant cells have a higher ability to perform glycolysis and have a higher capacity to increase glycolysis when mitochondrial energy production is compromised. (**C**) OCR:ECAR ratio for C200 and A2780 cells, demonstrating bioenergetic phenotypes for each cell type. (**D**) 2-deoxyglucose decreases ECAR in A2780 and C200 cells, while OCR increases compared to basal levels in C200 cells, indicating their ability to adapt in the presence of a glycolysis inhibitor. (**E**) Oligomycin decreases OCR in A2780 and C200 cells, while ECAR increases, compared to basal levels and chemosensitive cells, in C200 cells, further indicating their ability to adapt in the presence of a mitochondrial respiration inhibitor. *** *p* < 0.001, ** *p* < 0.01, * *p* < 0.05. Adapted with permission from Dar et al., Scientific Reports; published by Nature Portfolio, 2017. Creative Commons License: http://creativecommons.org/licenses/by/4.0/ (accessed on 9 February 2023) [19].

In another study, Dar et al. [19] examined the relationship between ovarian cancer cell bioenergetic profiles and phenotype in 13 ovarian cancer cell lines. The investigators found that bioenergetic profiles differed greatly between the cell lines, but enhanced capacities for glycolysis and OXPHOS were associated with increased proliferation and oxidative stress. Additionally, when comparing chemosensitive and chemoresistant cells, C200 cells, which are chemoresistant, had elevated basal OCR and ECAR, respiratory and glycolytic capacities, and respiratory and glycolytic reserves compared to the A2780 chemosensitive cell line (Figure 4A,B) [19]. According to the authors, this suggests that chemoresistant cells have highly metabolically active profiles compared to chemosensitive cells, which relied much more on glycolysis for energy production (Figure 4C). Furthermore, compared to chemosensitive cells, chemoresistant cells were better able to adapt after bioenergetic stresses. For example, Dar et al. [19] showed that 2-deoxyglucose exposure decreased ECAR in A2780 and C200 cells. However, C200 cells were able to adapt by increasing OCR compared to A2780 cells (Figure 4D). Similar findings were reported when A2780 and C200 cells were treated with oligomycin, as OCR decreased in both cell lines while ECAR increased in C200 cells compared to baseline (Figure 4E). While both studies suggest that bioenergetic profile-targeted therapies may be promising in the treatment of ovarian cancer, significant challenges may arise due to the differing bioenergetic profiles across ovarian cancer subtypes. Determining which bioenergetic profile to target is cancer-specific and requires cancer-specific biomarker identification. 

A less time-consuming and costly alternate approach to measuring OCR and ECAR is the MitoXpress-Xtra^®^ assay (Agilent) and pH-Xtra™ assay (Agilent), respectively. Compared to the Seahorse Extracellular Flux Analyzer, these assays are simpler in that endpoints are measured using a time-resolved fluorescence plate reader. The pH-Xtra™ assay measures ECAR using a fluorescent lanthanide probe while the MitoXpress-Xtra^®^ assay measures OCR using a phosphorescent probe [268]. It is important to note that seeding density must also be optimized for these assays prior to obtaining bioenergetic measurements. Rather than injecting glycolytic or ETC substrates/inhibitors, bioenergetic measurements of cells exposed to these compounds are performed in separate wells of a 96-well plate [268]. Study-related exposures are added to the plates, followed by addition of the MitoXpress-Xtra^®^ or pH-Xtra™ reagent immediately prior to measuring OCR and ECAR, respectively.

While Seahorse analyzers are gaining popularity, mitochondrial bioenergetics, specifically respiration, can be measured using other techniques as well. One method is the Orosboros 2k-Oxygraph system (Orosboros Instruments, Innsbruck, Austria). This high resolution respirometry system enables real-time measurements of respiration while eliminating unstable signals and background noise that are common in other respirometry techniques, such as the Clark electrode [275,276,277]. For this technique, airtight reaction chambers and oxygen detecting sensors are used in conjunction with Oroboros Datlab software to determine the bioenergetics of isolated mitochondria [277,278]. Like the Seahorse analyzer and microplate assay protocols, mitochondrial complex inducers or inhibitors are used to evaluate changes in respiration in isolated mitochondria. Although the Orosboros 2k-Oxygraph is a somewhat newer technology, it has been used to measure respiratory changes in a variety of different cancer cell types. For example, a study evaluating mitochondrial metabolic reprogramming in high-grade prostate cancer used Orosboros to perform high resolution respirometry. Compared to benign tissues, malignant tissues demonstrated increased succinate oxidation and succinate-fueled OXPHOS [279]. Respirometry after various chemotherapy or antibody-based treatments has also been evaluated in cancer cells using Orosboros. In skin cancer cells, treatment with imiquimod led to decreased oxygen consumption compared to untreated keratinocytes [280]. Additionally, using synthetic isoflavan analogues that have been shown to inhibit tumor proliferation and metastasis, complex I activity levels decreased in 143B osteosarcoma and HeLa cells [281].

A traditional and reliable method for measuring oxygen consumption is using a Clark electrode coupled to polarography [282]. In this approach, a closed oxygen consumption chamber is filled with respiration buffer prior to the addition of isolated mitochondria [283]. Using a monitor with a catalytic platinum surface within the system, changes in oxygen consumption after the addition of isolated mitochondria and ETC complex inducers or inhibitors are measured [282]. Oxygen consumption reads are typically coupled to lab analysis software to understand changes in atomic oxygen in the chamber over time [283]. It is important to note that measurements of bioenergetics can be performed on isolated mitochondria from intact cells or permeabilized cells. Mitochondrial isolation can reduce both yield and quality, so performing analyses of bioenergetics on permeabilized cells circumvents this issue [284]. Since the Clark electrode is a reliable method for evaluating oxygen consumption, many studies have used this technique to measure changes in respiration in cancer cells before or after treatment with chemo- or radiotherapy. In a study by Elbaz et al. [285], treatment of pancreatic cancer cells with the flavonoid epicatechin stimulated mitochondrial oxygen consumption, thereby sensitizing cells to radiation therapy. The Clark electrode was also used in studies evaluating ovarian cancer progression and response to therapy. In one study, the Clark electrode was used to measure respiration in ovarian cancer patient-derived xenograft ascites cell pellets. Results demonstrated that increased respiration alongside high expression of PGC1α and PGC1β were associated with malignant progression. Additionally, respiration and mitochondrial health associated with malignant progression decreased upon treatment with a complex I inhibitor (IACS-010759) [286]. In another study evaluating platinum-sensitive ovarian cancer cells (A2780) treated with Auranofin, a drug commonly used to treat rheumatoid arthritis, the drug was found to impair OXPHOS and shift the cell towards glycolysis for energy production [287]. 

Yet another method for measuring oxygen consumption is electron paramagnetic resonance (EPR) oximetry. EPR oximetry is minimally invasive and can measure oxygen levels both in vitro and in vivo [288,289]. Studies have demonstrated the effectiveness of using this technique to monitor ischemic or hypoxic tissue oxygenation [288,290,291,292,293]. The underlying principle of EPR oximetry is that electrons with unpaired spins absorb radiation and move from low to high energy levels when exposed to magnetic fields [288]. To perform this technique, a paramagnetic external spin probe collides with molecular oxygen to induce dipole–dipole interactions and Heisenberg spin exchange which in turn induces changes in EPR linewidth [288]. A change in EPR linewidth enables the measurement of oxygen concentration. Interestingly, this technique can be used on tumor cell suspensions, isolated perfused organs, or living tissue [288], and longitudinal measurements over days or weeks are possible. While this technique is promising for the clinical detection of tissue oxygenation during various disease states, there are quite a few drawbacks to using EPR oximetry, including poor signal-to-noise ratios and motion artifacts [288]. 

Since there are many methods to characterize mitochondrial bioenergetics, a study by Diepart et al. [294] compared the readouts of OCR using EPR oximetry, the Clark electrode, and MitoXpress^®^ assay. In K562 lymphoblast cells, EPR oximetry was the most sensitive method to detect changes in tumor cell OCR. EPR oximetry was able to detect changes in oxygen after the addition of either rotenone or hydrocortisone [294]. The Clark electrode and MitoXpress^®^ assay were only able to detect effects after the addition of rotenone, and therefore were less sensitive in this cell type. In summary, Table 3 demonstrates the strengths and limitations of the various techniques used to define mitochondrial bioenergetics described here.

#### Measures of ETC Complex Activity and the TCA Cycle

When determining changes in mitochondrial bioenergetics, understanding functional and structural alterations in ETC complexes or supercomplexes may narrow down targets for potential therapeutics. Supercomplexes form when individual ETC complexes aggregate to form high-order stoichiometric combinations [299]. To measure ETC complex and supercomplex activity, blue or clear native polyacrylamide gel electrophoresis (BN-PAGE or CN-PAGE, respectively) can be used [299,300,301,302,303]. Since both individual complex and supercomplex activity have been measured simultaneously via BN- or CN-PAGE, these complexes co-exist, although the impact of supercomplexes on mitochondrial function remains poorly understood [299]. It is thought that supercomplexes can provide advantages, such as enhanced electron transport efficiency and decreased electron leakage. However, deleterious effects to one complex may affect the remainder of aggregated complexes [304,305,306,307,308,309]. 

Compared to sodium dodecyl sulfate (SDS)-PAGE, BN- or CN-PAGE preserves protein–protein interactions, which is critical for assessing supercomplex activity [299]. To perform BN-PAGE, mitochondrial solubilization and centrifugation are required prior to the addition of Coomassie G-250 anionic dye. Alternatively, membranes or organelles can be isolated using centrifugation, then suspended in a buffer with a carbohydrate to protect complex integrity [310]. Coomassie G-250 binds membrane proteins, more specifically inner membrane complexes, and induces a charge shift on bound proteins that leads to the separation of proteins by size [301].

After performing BN-PAGE to separate ETC complexes, follow-up assays can be performed to further study ETC complex structure and function including immunoblotting, in-gel assays, and purification by electroelution [300,310]. Immunoblotting on BN-PAGE gels can be difficult due to the interference of antibody detection by Coomassie G-250 [300]. Nonetheless, immunoblotting can be performed using antibodies targeted against specific proteins associated with complexes I–V. Depending on the antibody used, the presence of specific ETC complexes and related supercomplexes can be quantitated from the resulting immunoblot [310]. In-gel assays can be used to determine the dependence of supercomplexes present in the inner membrane on complex-specific activity. For example, BN-PAGE gels can be used to evaluate complex I activity by incubating gels with NADH and nitroblue tetrazolium, or complex V activity by incubating the gel with oligomycin, a complex V inhibitor [300]. In gels probed for complex I activity, all complex I-containing complexes and supercomplexes turn blue due to the reaction between electrons transferred from NADH to nitroblue tetrazolium. Conversely, since oligomycin is an inhibitor of complex V, all bands in the gel containing complex V monomers, dimers, or supercomplexes are eliminated. ETC complex or supercomplex activity can also be measured using electroelution, where only one band of interest from the gel is excised and proteins from this portion are purified via elution [300]. This involves running the excised eluate on another gel prior to staining and immunoblotting against a complex-specific proteins. 

Using these techniques, studies have evaluated the role of individual mitochondrial ETC complexes and supercomplexes in various types of cancers. Rai et al. [311] reported inhibited complex I activity, resistance to complex I-mediated oxidative stress, and impaired mitochondrial function in colorectal cancer cell lines with low metastatic potential. Normal complex I activity was observed in colorectal cancer cells with high metastatic potential, but inhibition of complex I in these cells decreased metastatic signaling and increased cell death [311]. In a study evaluating breast and endometrial tumorigenesis, Ikeda et al. [312] reported that supercomplex assembly, measured via BN-PAGE and immunoblotting, not only promoted tumorigenesis of both cell types through inducing metabolic alterations, but also enhanced tumor cell tolerance to hypoxic environmental conditions. In the context of ovarian cancer, BN-PAGE coupled with immunoblotting has been used to show that complex I impairment leads to an increase in PGC1α, the main regulator of mitochondrial biogenesis that controls oxidative metabolism and promotes the expression of OXPHOS complexes [313,314]. 

While techniques to measure OCR, ECAR, and complex activity are generally well-established, a novel method to measure TCA cycle flux was recently published by Bartman et al. [315]. In this study, glycolytic and TCA cycle flux were evaluated in mouse tumor tissues using isotope tracing and mass spectrometry, and in five viable solid tumor types, TCA flux was lower compared to that of healthy tissues. Interestingly, TCA flux increased by threefold compared to healthy controls in a mouse model of lymphocytic leukemia, suggesting that solid tumors behave different than hematologic malignancies. Despite solid tumors having lower TCA flux compared to healthy tissues, metastatic nodules in triple negative breast cancer mouse models had higher TCA flux than surrounding tissues. To examine the discrepancy between TCA flux in solid tumors vs. metastatic tissues, Bartman et al. [315] evaluated ATP-consuming tasks in both tissue types and found that solid tumors downregulate ATP-intensive processes, such as fat digestion and gluconeogenesis, potentially to spare ATP for proliferation. This study provides a novel method for evaluating TCA flux as well as insight into the role of bioenergetics in cancer progression. 

## 4. Conclusions

The mitochondrion is a key regulator of major cellular processes, and structural or functional alterations are associated with a variety of diseases including cancer. Genetic mutations and environmental factors, such as exposure to chemical contaminants, can contribute to mitochondrial dysfunction. Regardless of whether genetic or environmental stimuli are responsible, mitochondrial dysfunction, specifically deregulated bioenergetics, is considered a hallmark of cancer [8]. Recent studies suggest that increased dysregulation of mitochondrial function is correlated with increased tumor aggressiveness and resistance to therapy, highlighting the need for mitochondrial-targeted therapies. 

To effectively target mitochondria in the disease process, it is critical to understand the mitochondrial processes that are disrupted. For example, studies have shown that in ovarian cancer cells, enhanced bioenergetic flexibility, the ability to switch between OXPHOS and glycolysis for energy production, contributes to tumor cell survival and chemotherapy resistance [19]. Chemotherapy resistance is a main contributor to the high lethality of ovarian cancer. Thus, developing mechanism-based modalities that target and overcome resistance may improve patient outcomes. Alterations in mitochondrial structure have also been shown to underlie aggressive tumor phenotypes in difficult-to-treat cancers. In colon carcinoma cells, an interconnected network of mitochondria was observed in doxorubicin-sensitive cells, whereas doxorubicin-resistant cells displayed highly fragmented mitochondria. Doxorubicin-resistant cells also displayed enhanced bioenergetic capacities [70], highlighting how structural alterations may inform functional parameters underlying disease. 

Structural parameters of mitochondrial health covered in this review include changes in cristae morphology and mitochondrial dynamics (fission and fusion), mtDNA content and integrity, and EV secretion and content. Morphologic changes in mitochondria are often evaluated using microscopy, and these techniques can broadly be grouped into optical and electron-based methods. Generally, optical microscopy requires fluorescent mitochondrial markers in live or fixed cells and includes confocal microscopy, super-resolution techniques, and TPEF. Since the size of mitochondria is near the resolution limit for optical microscopy, EM may be more advantageous for evaluating ultrastructure. EM techniques, such as TEM, electron tomography, and cryo-SXT, can be used to visualize mitochondrial pathological morphologies and provide 3D maps of structural compartments. 

In addition to microscopy-based approaches, quantification of mtDNA content can provide insight into functional parameters, such as energy production. MtDNA is most often quantified using qPCR, but HPLC and gel electrophoresis coupled to Southern blotting can also be used. More recently, dPCR has been used to evaluate mtDNA copy number variation and mtDNA mutations or deletions, which may help identify therapeutic targets for cancer treatment. The final structural parameter discussed was EV secretion and content. EVs can contain genetic material and even organelles, so assessing content and secretion is important to understanding mitochondrial functional changes. EVs can be isolated using ultracentrifugation followed by NTA, Western blotting, or miRNA analysis, all of which can identify potential biomarkers related to proliferation, metastasis, and therapy response. It is important to note that while structure and function are discussed separately in this review, the relationship between the two is critical for understanding mitochondrial dysfunction and disease.

Compared to structural parameters, functional parameters of mitochondrial health are more likely to be targets of therapeutic intervention. These include ROS production, calcium signaling and retention, ΔΨ_m_, and bioenergetics. Oxidative stress profiles in cancer cells are critical since ROS-induced apoptosis is the mechanism of action of many chemotherapeutics. Elevated baseline levels of ROS, and antioxidants, in cancer cells can therefore diminish chemotherapeutic efficacy, enhancing tumor cell survival. ROS production can be measured using commercially available fluorescence-based assays or via OxyBlot, which is a more complex technique, but more specific and quantitative than plate reader assays. ΔΨ_m_ is considered a proxy of mitochondrial health since membrane damage is generally detrimental and alters normal functioning. Using fluorescent dyes compatible with microscopy, microplate spectrophotometry, or flow cytometry, ΔΨ_m_ can be evaluated at baseline and following experimental treatment. Decreases in ΔΨ_m_ are often observed in cells undergoing apoptosis, whereas increased ΔΨ_m_ can signify enhanced energy production and improved mitochondrial health. Similar to ΔΨ_m_ and ROS production, calcium signaling and retention, which play key roles in ATP production and apoptosis, can be measured using commercially available fluorophores. Aberrant calcium signaling often occurs in cancer, leading to enhanced proliferative and angiogenic capabilities. One functional parameter that therapeutics have attempted to target, but have yet to be successful, is bioenergetic capacity. In cancer cells, bioenergetic flexibility is often enhanced, meaning cancer cells can use glucose or oxygen for energy production depending on which substrate is more readily available. 

Although the methods outlined in this review can be useful for understanding mitochondrial structural and functional changes in disease models, it is important to note that these techniques are largely limited to cell-based models. Some methods discussed in this review are applicable in vivo, or tissues harvested from in vivo models. For example, mtDNA, EV content, and bioenergetics can all be measured using harvested in vivo samples, e.g., biofluids or tissues. TPEF and EPR oximetry can be used in vivo in real-time, allowing potentially more accurate readings of mitochondrial health or functioning. Additionally, TPEF and select methods for measuring energy production allow longitudinal evaluations of the same sample. This is advantageous because it only requires limited resources (one sample), but also because changes in mitochondrial function can be monitored over the course of an exposure period or treatment. 

Evaluating mitochondrial health and disease in human patients is complex and most methods are not compatible. Methods that are compatible require a liquid biopsy or biomatrix and remain challenging to use for real-time measurements. Patient tissue samples are compatible with some of the techniques described but cannot capture mitochondrial health in real-time. EPR oximetry, TPEF, and EVs have promising clinical potential. However, these techniques are in the beginning stages of human application. While approaches in humans are currently lacking, evaluating mitochondrial structural and functional changes in cell-based and animal models may lead to the identification of novel therapeutic targets for the treatment of diseases where mitochondrial health is known to be altered, including in cancers. 

## Figures and Tables

**Table 1:** Strengths and limitations of different microscopy types in the context of mitochondrial status.

Method	Applications	Strengths	Limitations	Ref
Widefield fluorescence and confocal microscopy	Mitochondrial morphology and dynamics, ΔΨm	Live-cell and time-lapse imaging Visualization of fluorescent proteins, dyes, and immunofluorescence staining	Resolution limited by diffraction (~1/2 λ) Fluorophore photobleaching	[25,78,79]
Super-resolution microscopy (STED, FPALM, STORM, etc.)	Fine details of mitochondrial morphology and dynamics (e.g., cristae shape, width, etc.)	Superior resolution compared to the confocal Live-cell and time-lapse imaging	Requires specific fluorophores Often needs high laser powers	[33,34,35,39,80]
Two-photon excitation fluorescence (TPEF) and fluorescence lifetime imaging	Mitochondrial dynamics, morphology and label-free redox state measurements, in vivo applications	Low background Superior light penetration depth Live-cell and time-lapse imaging Visualization of external probes or endogenous metabolites	Limited number of external fluorophores suitable for two-photon excitation	[44,45,81,82]
Electron microscopy (EM)	Ultrastructural changes in mitochondrial shape, size, and components	Sub-nanometer resolution	Requires extensive fixation Limited ability to visualize markers Prone to artifacts	[58]
Electron tomography	3D information on mitochondrial ultrastructure	3D structural information with high resolution	Requires extensive fixation “Missing wedge” because of the restricted tilt range Sample shrinkage due to high electron dose Limited ability to visualize markers	[65,83,84,85]
Focused ion beam scanning electron microscopy (FIB-SEM)	3D structural and compositional analysis	Label-free Resolution approaching EM	Not suitable for live cells Long image acquisition time (up to 60 h)	[71,72,73,74,75,76,77]

**Table 2:** Strengths and limitations of different PCR methods.

Method	Strengths	Limitations	Refs
Quantitative PCR	High throughput High sensitivity and specificity Established methods Simply and widely used Real-time monitoring of target Versatile	Reliant on standard curves or normalization to reference gene Prone to PCR efficiency bias Sensitive to PCR inhibitors Sample quality requirements	[117,118,119]
Digital PCR	High throughput Highly sensitive Absolute quantification Improved reproducibility Reduced PCR efficiency bias and PCR inhibition Highly accurate for low target concentrations No need for reference genes	Specialized equipment needed Cost prohibitive Extensive optimization required Sample quality requirements Narrow dynamic range Less accurate for high target concentrations	[117,118,127,128]

**Table 3:** Strengths and limitations of different methods for measuring oxygen consumption.

Method	Strengths	Limitations	Refs
Seahorse Extracellular Flux Analyzer	High throughput Requires small quantities of cells Automated process/injections Compatible with broad range of cell and tissue types	Costly equipment setup Moderate optimization required Significant plate and reagents costs per experiments	[271,284,295]
MitoXpress-Xtra^®^	Microplate-based assay High throughput Compatible with broad range of in vitro models Compatible with many plate readers	Moderate optimization required Significant plate and reagent costs per experiment Lower sensitivity Less well-established	[268,294]
Orosboros-2k Oxygraph	Relatively cheap Low sample volumes required Reduced oxygen leakage from device compared to other electrodes Increased sensitivity compared to other electrodes	Measurements are not automated Labor-intensive Low throughput Time-intensive (hour-long sample reads) Lacks background controls	[277,296]
Clark Electrode	Well-established method Reliable Mechanically robust Relatively low cost	Consumes oxygen Interference with various gases Labor-intensive maintenance	[294,297,298]
EPR Oximetry	Higher sensitivity Relatively non-invasive Enables 3D oxygen mapping Minimal interference issues Spin probes are non-toxic & stable	Poor signal-to-noise ratio Motion artifacts Requires exogenous probe Longer acquisition times Limited penetration depth	[288,294]

## Data Availability

As this is a review, no original data are presented in this study.
